# Electrospun Silver Coated Polyacrylonitrile Membranes for Water Filtration Applications

**DOI:** 10.3390/membranes8030059

**Published:** 2018-08-08

**Authors:** Shalv Amit Parekh, Rebecca Nicole David, Kranthi K. R. Bannuru, Lakshminarasimhan Krishnaswamy, Avinash Baji

**Affiliations:** 1 Engineering Product Development, Singapore University of Technology & Design, Singapore 487372, Singapore; shalv_parekh@mymail.sutd.edu.sg (S.A.P.); rebecca_nicole@mymail.sutd.edu.sg (R.N.D.); kranthi_bannuru@mymail.sutd.edu.sg (K.K.R.B.); l_krishnaswamy@sutd.edu.sg (L.K.); 2 Department of Engineering, School of Engineering and Mathematical Sciences, La Trobe University, Bundoora, VIC 3086, Australia

**Keywords:** polyacrylonitrile, electrospinning, antibacterial, silver nanoparticles, water filtration

## Abstract

The scarcity of drinking water and the contamination of water sources in underdeveloped countries are serious problems that require immediate low-tech and low-cost solutions. In this study, we fabricated polyacrylonitrile (PAN) porous membranes coated with silver nanoparticles (AgNP) and demonstrated their use for water filtration and water treatment applications. The membranes were prepared by electrospinning a PAN solution and treating in a hydroxylamine (NH_2_OH) aqueous solution to form –C(NH_2_)N–OH groups that were used for functionalization (Ag^+^ ions) of the membrane. The coordinated silver ions were then converted to silver nanoparticles. The microstructure of the membrane, water permeability, antimicrobial effect (using *Escherichia coli*), and particulate filtration capabilities were studied. This study verified that the membrane demonstrated a 100% reduction for Gram-negative bacteria with an effective filtration rate of 8.0 mL/cm^2^ min. Furthermore, the membrane was able to eliminate 60% of latex beads as small as 50 nm and over 80% of the 2 µm beads via gravity filtration. This study demonstrated that PAN–AgNP membranes can be employed as antimicrobial membranes for the filtration of water in underdeveloped countries.

## 1. Introduction

Water shortage and the availability of clean drinking water sources are of serious concern in most developing and developed countries. To address these issues, researchers have utilized porous membranes for developing filter media that can be used for water purification and water treatment. Various polymers have been used to fabricate electrospun membranes, such as polyacrylonitrile (PAN), polysulfone (PSU), polyethersulfone (PES), polyvinylidene fluoride (PVDF), polypropylene (PP), and cellulose acetate [1,2,3,4,5,6,7,8,9,10,11,12,13,14,15]. PAN has excellent properties, including high mechanical properties, a unique thermal stability, and an ability to undergo pre-spinning and post-spinning modifications [2,3,4,5,6,9,12,13,14,16,17,18,19,20,21,22,23,24,25,26,27,28]. Furthermore, PAN porous membranes produced using various processing techniques have been widely investigated for water filtration applications [1,2,3,4,5,6,7,8,9,10,11,12,13,14,15,16,17,18,19,20,21,22,23,24,26,29,30]. Compared to other conventional processing techniques, electrospinning has obtained tremendous interest among researchers as the process is simple, reproducible, cost-effective, and scalable for the development of porous membranes for water filtration and treatment [2,3,4,5,7,8,10,11,15,18,19,20,26,27,28,29]. Additionally, PAN membranes fabricated using electrospinning can be functionalized using various chemicals to make them anti-bacterial and anti-microbial [1,4,5,7,8,9,10,11,12,14,16,18,21,22,26,27]. Such membranes can potentially be used for water filtration in underdeveloped countries to combat the growing need for an affordable and effective filtration method to provide clean and safe drinking water.

Recently, silver nanoparticles have been incorporated in membranes for water filtration due to their anti-microbial property [4,5,7,11,14,15,16,21,22,23,24,27]. In this study, we use electrospinning to fabricate silver nanoparticles (AgNP) coated PAN membranes and demonstrate their use for water filtration and treatment. Previous studies have shown that antimicrobial agents such as silver can be incorporated into the fibrous membrane by simply dispersing them into the electrospinning solution prior to electrospinning. However, it has been reported that the incorporation of antimicrobial agents within the fibre matrix may not impart antibacterial properties evenly in the membrane [6,13,22,23,30]. In this study, we first used electrospinning to fabricate PAN fibrous membrane and then coated the surface of the fibres with silver particles. This step enabled a higher retention of silver nanoparticles on the membrane compared to dispersing silver particles within the electrospinning polymer solution [6,13,16,20,22]. The membranes were investigated for antimicrobial efficacy against *Escherichia coli* bacteria [2,3,4,6,8,9,10,11,12,14,15,16,24,26,27,30,31], water permeability, and particulate filtration capabilities using amine-modified blue fluorescent latex beads [4,19]. The results indicated that the membranes possessed antimicrobial functionality to kill bacteria and stop their colony growth. Water permeability test is used to investigate the water filtration rate of the membranes. Furthermore, the particulate filtration test showed that the membranes can separate an acceptable number of particles with sizes as small as 50 nm diameter. This demonstrates their ability to filter contaminant particles from the water.

## 2. Materials and Methods

### 2.1. Materials

The polyacrylonitrile (PAN) (Mw: 150,000) used in this study was obtained from Scientific Polymer Products, Inc. (Ontario, NY, USA). *N*,*N*-dimethylformamide (DMF) was purchased from TEDIA (Fairfield, OH, USA) . Other materials, such as acetone, hydroxylamine (NH_2_OH), and silver nitrate (AgNO_3_) were purchased from Sigma Aldrich (Singapore).

### 2.2. Solution Preparation

The electrospinning solution was prepared by dissolving 16 wt.% PAN in DMF. The solution was heated at 70 °C for 8 h to ensure the complete dissolution of PAN.

### 2.3. Electrospinning

**Figure 1:**
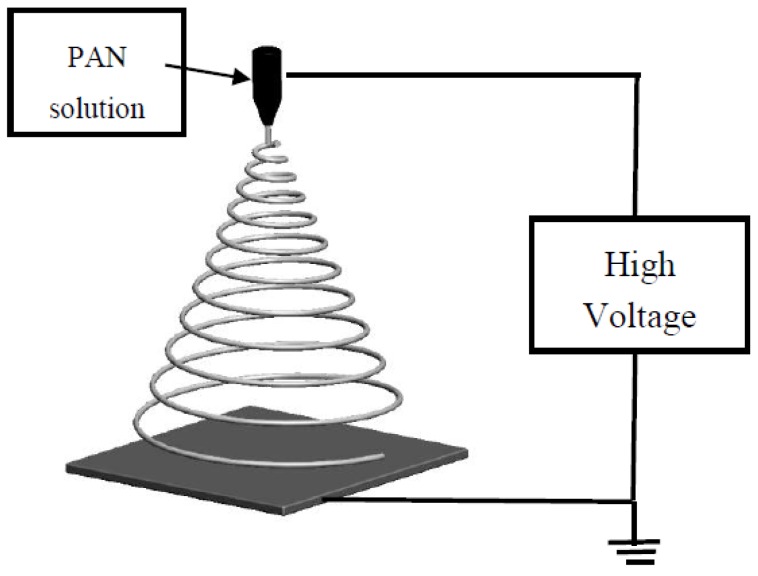
Schematic of electrospinning setup used to prepare the membranes. PAN: polyacrylonitrile.

The freshly prepared solution was filled in a 10 mL syringe with a 21-gauge Terumo stainless-steel flat round tip needle. A schematic of the electrospinning set up is shown in Figure 1. A rotating drum with aluminium foil was used as a collector, at a distance of 15 cm from the spinneret. A voltage of 15 kV was applied to the needle using a built-in voltage source, and the flow rate for the solution was 0.5 mL/h, maintained using a syringe pump. The controlled environmental conditions were set at a temperature of 25 °C and relative humidity of 75%. A 10 × 10 cm^2^ PAN membrane was fabricated using 30 mL of the solution. The aluminium foil containing the membrane was removed and dried in an oven overnight at 60 °C. 

### 2.4. Surface Functionalization

The following steps were used to coat the surface of fibres with silver particles. In the first step, the electrospun PAN membrane was completely immersed in 1 M NH_2_OH for 20 min. This was followed by an immersion in 0.1 M AgNO_3_ aqueous solution at 25 °C for 1 h to allow the amidoxime groups from NH_2_OH to coordinate with the silver ions (Ag^+^). The prepared membrane was then immersed in 0.01 M KBr aqueous solution for 2 h, followed by a quick immersion in methanol. Finally, the membrane was exposed to ultraviolet (UV) for 10 min on each side in the Triad 2000 chamber (DENTSPLY International Inc., York, PA, USA). The membrane was then thoroughly washed in distilled water and left to dry in the oven at 70 °C for 7 h followed by vacuum drying. 

### 2.5. Characterisation

A scanning electron microscope (SEM, JSM-6700F, JEOL, Tokyo, Japan) was used to investigate the morphology of the PAN and PAN–AgNP membranes. Fourier-transform infrared spectroscopy (Vertex 70, Bruker, Karlsruhe, Germany) was utilised to study the different chemical bonds present in the AgNP-coated and uncoated PAN membranes. The study was done in the Attenuated Total Reflection (ATR) mode. The lattice structures of the samples were studied by X-ray powder diffraction (XRD, Bruker D8 Advance diffractometer, Bruker Taiwan) with Cu Kα (λ = 0.154 nm) radiation at an accelerating voltage of 40 kV.

### 2.6. Antimicrobial Tests

The antimicrobial effect of the PAN membranes with and without silver nano-particles was tested on *E. coli* cells derived from Top10 cells (Thermo Fischer Scientific, cat C404010 Singapore) as a representative of Gram-negative bacteria. *E. coli* was cultured in Luria-Bertani broth (LB, Carolina Biological Supply Company, cat.216650, Burlington, NC, USA) in an orbital shaker incubator (YIHDER Technology, LM 510-RD, Xinbei City, Taiwan) at 37 °C, 120 rpm for 24 h. The bacteria were harvested by centrifuge, washed with 1 X PBS (phosphate-buffered saline) and then re-suspended in 1 X PBS. PBS was prepared by mixing 1 part of 10 X PBS (from Bio-Rad Laboratories, Inc, cat.1610780, Hercules, CA, USA) with 9 parts of sterile ultrapure water. The density of *E. coli* was determined by spectrophotometry and adjusted to a density of 10^7^ cfu/mL (colony forming units/millilitre) using 1 X PBS. Then, 50 μL of this suspension of bacterial cells was placed on samples of the PAN membranes and PAN membranes with silver nano-particles (each 1 cm^2^), for 30 or 60 min. After the defined duration of bacterial contact with the membrane, the membrane was placed in 10 mL of 1 X PBS solution and vortexed for 2 min. This was done to transfer any remaining bacteria from the membrane to the 1 X PBS solution. Then, 50 μL of the 10^7^ cfu/mL bacteria solutions was directly placed in a separate 10 mL of PBS solution as a control. The 10 mL solutions were then diluted serially, and 100 μL of the diluent was plated onto LB agar plates (Carolina Biological Supply Company, cat. 216620, Burlington, NC, USA). The plates were incubated at 37 °C for 20 h, and the number of colonies in each plate was determined. The test was carried out in three replicates for repeatability. 

### 2.7. Water Permeability Test

The permeability of water through AgNP-coated and uncoated PAN membranes was determined using a syringe at a constant flow rate. A 1 × 1 cm^2^ membrane sample was cut and exposed under UV light for 5 min. The membrane was placed on the tip of a Terumo 5 mL syringe such that it allowed for an effective filtration area of 0.186 cm^2^. The test maintained a constant pressure throughout the experiment under gravity. A total of three membrane samples were tested thrice each, and the corresponding flow rate readings were recorded for each membrane.

### 2.8. Filtration Test

The PAN membranes were cut into 2 cm × 2 cm^2^ to fit onto the Terumo 5 mL syringe. A solution of 5 mL of 800 ppm aqueous solution of two different amine-modified blue fluorescent latex beads of mean particle size of 2.0 μm and 50 nm was pumped in the syringe. The solution was passed through the fabricated PAN membrane at a constant pressure of 50.3 kPa. The collected water sample was analysed using spectrophotometry. The results presented here are based on an average of three independent trials.

## 3. Results and Discussion

### 3.1. Morphology

**Figure 2:**
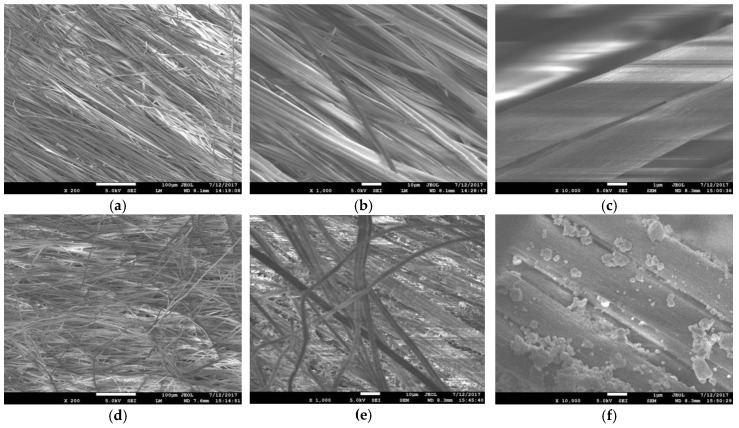
Scanning electron micrographs (SEM) of electrospun PAN fibres: (**a**) PAN at ×200 magnification (Scale Bar = 100 µm); (**b**) PAN at ×1000 magnification (Scale Bar = 10 µm); (**c**) PAN at ×10,000 magnification (Scale Bar = 1 µm); (**d**) PAN–AgNP (silver nanoparticles) at ×200 magnification (Scale Bar = 100 µm); (**e**) PAN–AgNP at ×1000 magnification (Scale Bar = 10 µm); (**f**) PAN–AgNP at ×10,000 magnification (Scale Bar = 1 µm).

**Figure 3:**
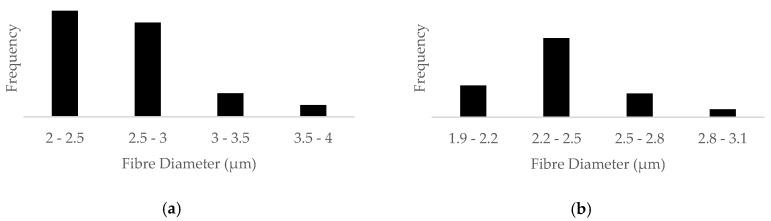
Histogram size distribution of: (**a**) PAN; (**b**) PAN–AgNP.

Figure 2 shows the SEM images of the PAN membranes before and after they are coated with silver particles. It is evident from the SEM images (Figure 2) that the PAN membranes are thick with many layers of fibres. The mean diameter of the fibers within the membrane is determined from these SEM images to be 2.36 ± 0.22 μm (Figure 3).

The incorporation of silver particles on the surface of the fibres is seen to make them hydrophilic. A goniometer is used to estimate the contact angle made by a water drop on these samples using the sessile drop technique. The initial contact angle of the PAN membrane is measured to be 19° ± 4°. On the other hand, the contact angle on silver coated PAN fibers is determined to be ~0°. This indicates that the silver coated membranes are superhydrophilic.

### 3.2. Structure

**Figure 4:**
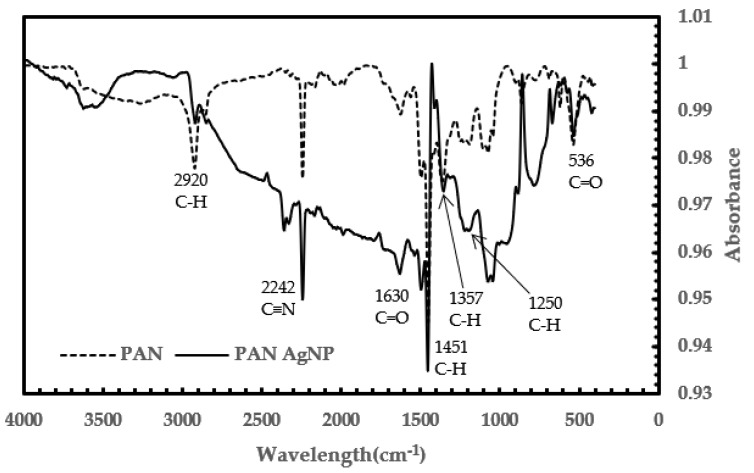
Comparison of Fourier transform infrared (FTIR) spectra of PAN and PAN with AgNP membranes.

Fourier-transform infrared spectroscopy (FTIR) is used to investigate the effect of adding silver on the surface of the PAN membrane. Most of the peaks in both samples are observed to be relatively similar, with some discrepancies. This showed that there is no noticeable change in the bonding in PAN and PAN–AgNP. Figure 4 shows the characteristic peak of PAN at 2242 cm^–1^ (assigned to C≡N), which showed no change in the C≡N bond with the addition of silver [2,3,19,23,25]. Thus, no coordination bond was formed between silver and PAN [3]. The peaks at 1451 cm^–1^ and 2920 cm^–1^ displayed the bending and stretching vibrations of the C–H_2_ functional group in PAN, respectively [2,19,23,25]. The peak at 1630 cm^–1^ assigned to C=O is due to the oxidation of PAN in air [2,23,25]. The peaks at 536 cm^–1^ and 1630 cm^−1^ are assigned to C=O twisting and C=O stretching [23]. The peaks at 1357 cm^–1^ and 1250 cm^–1^ are assigned to CH group vibrations of CH_2_ and CH, respectively [25]. With an increase in Ag, the peak intensity of C=O stretching should increase as well. This is due to the interaction between PAN and Ag with DMF [25]. 

**Figure 5:**
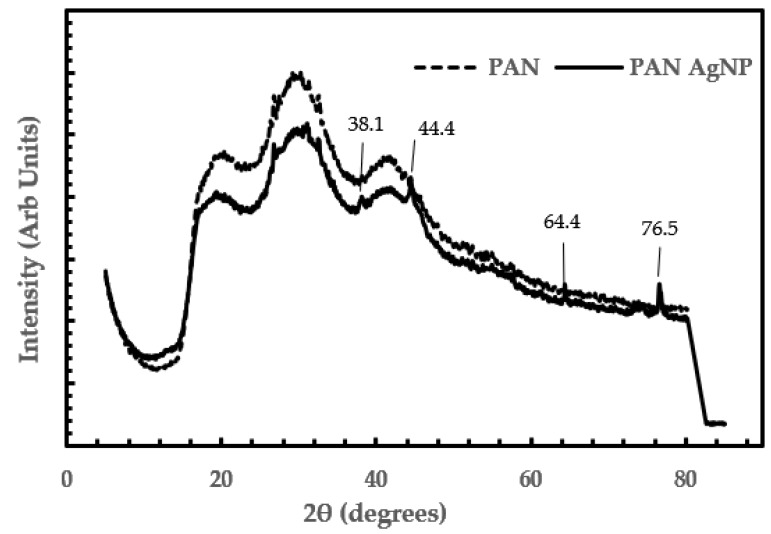
XRD plots of PAN and PAN–AgNP.

The XRD plots in Figure 5 are used for phase identification to determine the average bulk composition. The PAN microfibres coated with AGNP showed four distinct peaks, at 2θ values of 38.1°, 44.4°, 64.4°, and 76.5°. This showed the face-centred cubic (FCC) structure of silver in the silver coated PAN fibers with crystalline planes of silver (111), (200), (220), and (311), respectively [2,3,6,23]. The values we obtained are similar to the values found in the International Centre for Diffraction Data (ICDD) card (card no. 4-783). The XRD results are in agreement with the SEM and FTIR results.

### 3.3. Antibacterial Test Results

**Figure 6:**
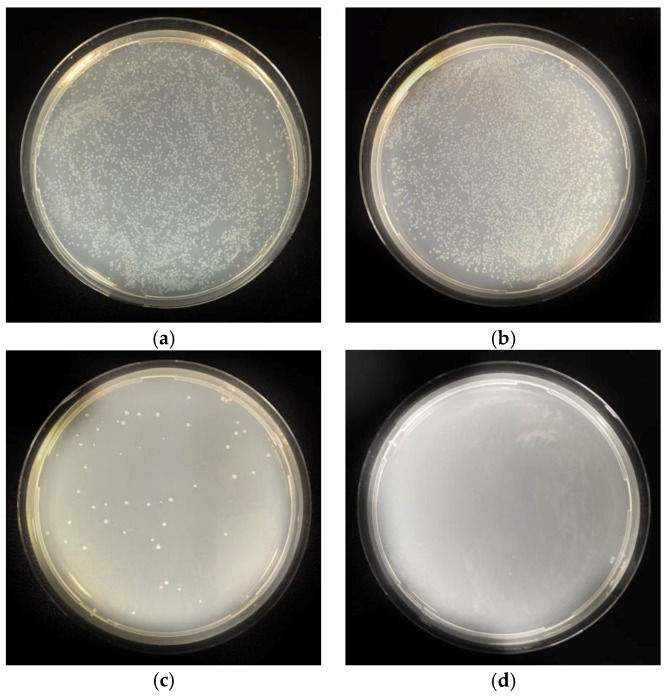
Agar plates of sample from the (**a**) Bacterial control; (**b**) PAN sample control; (**c**) PAN–AgNP sample (after 30 min); (**d**) PAN–AgNP sample (after 60 min).

Table 1 shows the antimicrobial performance of the silver coated PAN membranes. It is clear from Table 1 that there is a significant reduction in the number of bacterial colonies in the solution that is passed through PAN–AgNP membrane compared to the initial control solution. Similarly, the number of bacterial colonies in the solution that is passed through the silver coated PAN membrane is lower than in the solution that is passed through PAN membrane alone. Figure 6 show the digital images of the bacterial colonies on the Agar plates. It is clear that the presence of the AgNP on the membrane inhibited the growth of bacteria. The sample of the filter mesh killed over 99.99% of bacteria within just 30 min of contact (Figure 6c). Most of the samples had only one colony (Figure 6d), giving an average standard deviation between samples of 0.0045%.

### 3.4. Water Permeability Test Results

In order to confirm the potential application of the membrane in the filtration process, the permeability behaviour of the membranes is investigated. The water permeability is determined by measuring the flow rate of water through a 0.186 cm^2^ area of membrane with a constant pressure of 102,874 Pa across the membrane. The average values of water permeability from three trials are determined to be ~1.52 mL/min and ~1.49 mL/min for PAN membrane and silver coated PAN membrane respectively. This shows that the addition of silver particles did not alter the water permeability performance of the membranes. The normalized flow rates per unit area are determined to be 4902 ± 402 LMH and 4806 ± 372 LMH (LMH = Liters/M^2^/Hr) for neat PAN membrane and silver coated PAN membrane respectively. 

### 3.5. Fitration Test Results

**Figure 7:**
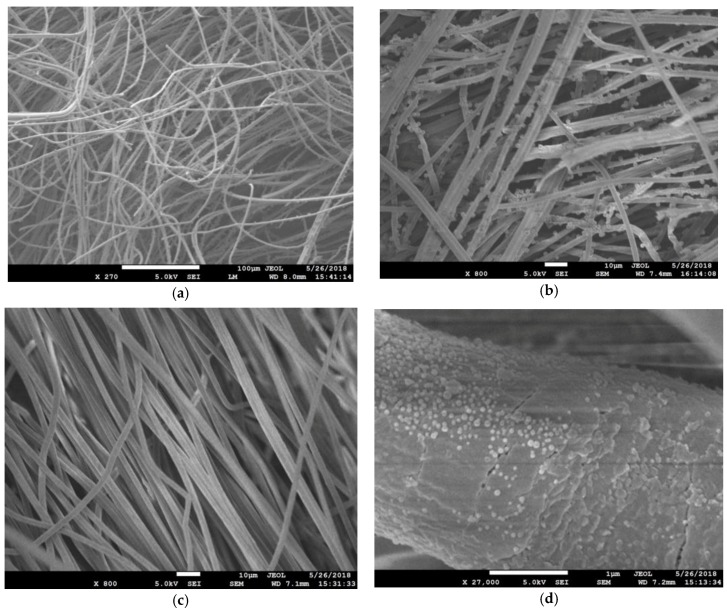
Filter membrane sample after filtration of solution with latex beads: (**a**) PAN with 2 µm latex beads at ×270 magnification; (**b**) PAN with 2 µm latex beads at ×800 magnification; (**c**) PAN with 50 nm latex beads at ×800 magnification; (**d**) PAN with 50 nm latex beads at ×27,000 magnification.

Following this, the PAN membrane is tested for particulate filtration efficiency using amine-modified blue fluorescent latex beads of 2 µm and 50 nm diameter. The results are displayed in Table 2. As expected, the membrane fibres captured the highest percentage of 2 µm latex beads. The capture efficiency is 81.2% for 2 µm beads and 61.4% for 50 nm beads filtered through the same membrane. The SEM images of membrane fibres post-filtration are shown in Figure 7. It can be seen that the 2 μm beads are significantly larger than the pore sizes, and are captured by the membrane’s fibre mesh (Figure 7a,b). In contrast, there is little evidence of the 50 nm beads on the fibre surface (Figure 7c), except for a few densely packed clusters on the fibres (Figure 7d). 

The membranes are reused to repeat the experiment three times to check for clogging. There was no significant drop in particulate filtration efficiency, suggesting that the filter membrane can be used multiple times.

## 4. Conclusions

Silver nanoparticle-functionalized PAN membranes were prepared by electrospinning followed by treating the membrane with hydroxylamine and AgNO_3_ by photoreduction. The resulting membrane had coordinated silver ions that were converted to silver nanoparticles. Immersion in hydroxylamine for 20 min and AgNO_3_ for 1 h resulted in an excellent anti-microbial membrane with total kill in 1 h. With a longer immersion in either or both solutions, the membranes have a potential for better antimicrobial properties. Furthermore, the water permeability test showed that the membrane displayed adequate water filtration capabilities for water filtration membrane applications. The filtration test also showed that despite our large fibre diameters, the membrane managed to filter over 61.4% of 50 nm particles and over 81.2% of 2 µm particles. This study thus proves the usability of PAN membranes with silver nanoparticles functionalization for water filtration purposes as an affordable and reliable solution for underdeveloped countries.

## Figures and Tables

**Table 1:** Antimicrobial efficacy of AgNP on PAN membranes tested on *Escherichia coli.*

Sample	Percentage Reduction	
	30 min	60 min
PAN (control)	No Reduction	No Reduction
*E. coli* (control)	No Reduction	No Reduction
PAN–AgNP Sample 1	99.997678	99.9995
PAN–AgNP Sample 2	99.989017	100.000
PAN–AgNP Sample 3	99.999213	100.000

**Table 2:** Particulate filtration efficacy of PAN membranes with latex beads.

Sample	Moles of Latex Beads (×10^5^)	
	Before Filtration	After Filtration
PAN with 2 µm	222.24	41.76
PAN with 50 nm	164.64	66.24
